# Building analytical capacity for research at Federally Qualified Health Centers through the *All of Us* Research Program

**DOI:** 10.1017/cts.2025.10142

**Published:** 2025-09-02

**Authors:** Soumya Kini, Derek Inokuchi, Jessica Burke, Jennifer Adjemian, Andrea Ramirez, Edgar A. Diaz, Grace Wang, Sarra Hedden

**Affiliations:** 1 The MITRE Corporation, McLean, VA, USA; 2 National Institutes of Health, Bethesda, MD, USA; 3 San Ysidro Health, San Diego, CA, USA; 4 Moses Weitzman Health System, Middletown, CT, USA

**Keywords:** Federally Qualified Health Center, *All of Us* Research Program, capacity building, precision medicine, biomedical research

## Abstract

The National Institutes of Health *All of Us* Research Program (*All of Us* or program) aims to better understand the complexity of diseases, prevention and treatment at the individual level. To accomplish this, one of the program components is to build a longitudinal cohort of one million or more volunteers in the United States and its territories through which clinical, environmental, genetic, and behavioral data are collected. Federally Qualified Health Centers (FQHCs) play a crucial role in enrolling participants in the program and while FQHCs have the dedication, leadership, and wherewithal to operationalize a national longitudinal data collection, their local resources are limited by funding and scope for conducting research. This paper describes the evolution of FQHC research landscape, from building capacity for descriptive, to exploratory operational research, and moving toward biomedical research. As programs such as *All of Us* continue to ensure that focus on precision medicine is reflected in both data collection and research, continuing to advance the research landscape within health centers is crucial. By developing this capacity, we are developing a research infrastructure that will continue to grow, supporting advancements in precision medicine for improving health outcomes.

## Introduction

The National Institutes of Health (NIH) *All of Us* Research Program (*All of Us* or program) initiative aims to better understand the complexity of diseases, disease prevention and the effectiveness of treatment at the individual level. To accomplish this, one of the components of *All of Us* is a longitudinal national research cohort of one million or more volunteers based in the United States (U.S.) and its territories through which data, including clinical, environmental, genetic, and behavioral data are being collected through surveys, electronic health records (EHRs), physical measurements, and biosamples. *All of Us* has created a comprehensive research resource, the Researcher Workbench (RW), accessible to researchers and the public with the purpose of improving the nation’s understanding of the prevention and treatment needs of individuals [1]. The RW is a cloud-based secure platform containing analytic tools with varying degrees of complexity that researchers can use to access data from the *All of Us* cohort (https://www.researchallofus.org/data-tools/workbench/)[2].

*All of Us* has established partnerships with Healthcare Provider Organizations, that include Regional Medical Centers (RMCs), Department of Veterans Affairs (VA) and Federally Qualified Health Centers (FQHCs) to achieve its enrollment and data collection goals in support of precision medicine [3,4]. FQHCs are part of the health center program at the Health Resources and Services Administration (HRSA). HRSA-funded health centers provide services to low-income, underserved, and uninsured individuals [5,6]. HRSA focuses on improving access to quality care and services, strengthening the health workforce, building healthy communities, and improving health outcomes nationwide. NIH, in collaboration with HRSA and in partnership with the Centers for Medicare and Medicaid Services’ Alliance to Modernize Healthcare Federally Funded Research and Development Center (Health FFRDC) - operated by the MITRE Corporation (MITRE), has engaged eight FQHCs across the country and U.S. territories in *All of Us* [7]. The partnership, called “central coordination,” is between NIH, MITRE, and the eight MITRE-managed FQHCs [5].

Since 2016, the MITRE-managed FQHCs have enrolled over 14,000 participants, retaining over 6,000 participants in the longitudinal cohort, employing over 70 full-time research staff, and publishing and presenting their research programs. The FQHCs’ success as *All of Us* enrollment partners is not without effort, as funding, resources and capabilities had to be built for FQHCs to become strong enrollment partners. These efforts contributed to ensuring the *All of Us* RW truly reflects data that supports precision medicine and that research discoveries benefit FQHC patients and their communities. While the *All of Us* FQHCs have the dedication, leadership, and wherewithal to operationalize a national longitudinal data collection, their local resources are limited by funding and scope because of which their capacity to utilize the RW to conduct research projects has been limited. MITRE with NIH has piloted a process to address this, through a partnership with FQHCs, with the aim of bridging the gap between FQHC resources and technical research capabilities. This paper describes the evolution of research landscape of MITRE-managed FQHCs, from building capacity for descriptive, to exploratory operational research, and moving toward biomedical research.

### Evolution of the FQHC research landscape

**Figure 1.**
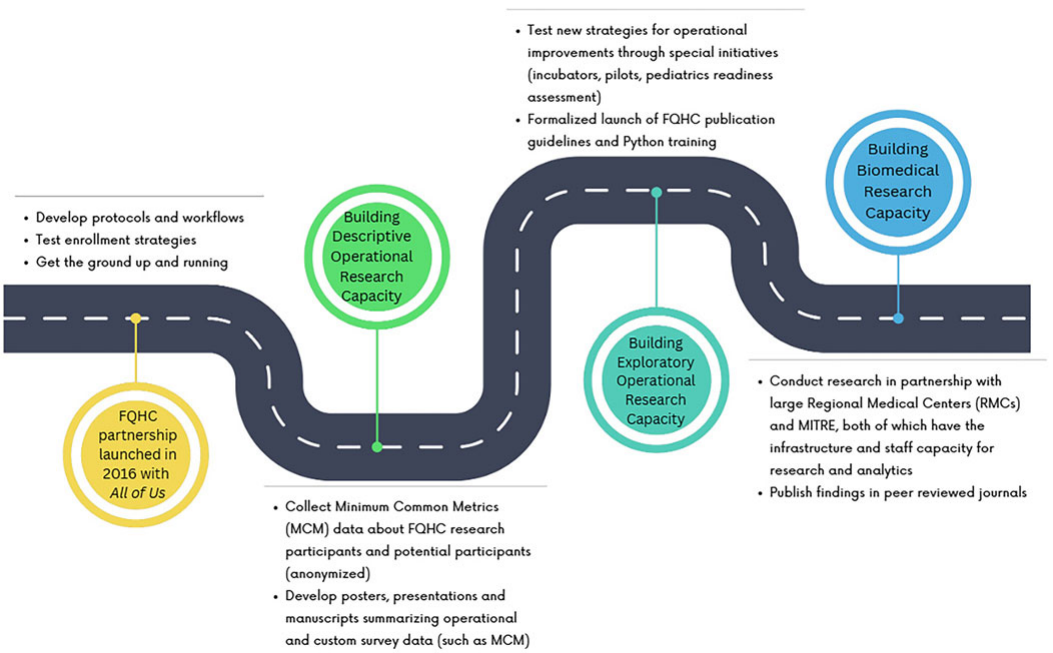
Evolution of the *All of Us* Federally Qualified Health Centers’ (FQHCs’) research landscape.

Figure 1 shows a timeline that illustrates the evolution of the FQHC research landscape in *All of Us*. Although some *All of Us* FQHCs started the program with more advanced research capabilities than others, the evolution can generally be divided into three main phases. The sections below provide more details on the three phases. Table 1 summarizes the distinction made in this paper between descriptive and exploratory operational research explained in the three phases below.

**Table 1.** Characterization of descriptive and exploratory operational research summarized in this paper

	Characterized by	Example research questions
Descriptive operational research	Examining frequencies and percentages of demographics, visits, timing, and survey responses	What is the proportion of male vs. female participants? What are primary motivators for joining the program?
Exploratory operational research	Evaluating the potential or actual impact of new strategies on program operations and outcomes	How does providing transportation services impact enrollment volume? What factors influence completion of ongoing program activities?

- Building capacity for “descriptive” operational research focused on creating or expanding on opportunities for FQHCs to collect their operational data and report on those metrics to MITRE and NIH.
- Building FQHC capacity for “exploratory” operational research continued the operational focus, but encouraged FQHCs to pilot or test new strategies that could improve their operational metrics, encouraging hypothesis driven research.
- Moving FQHCs toward biomedical research propelled FQHCs to leverage and analyze the data they collect from the program participants to develop and explore research topics that benefit their patients and communities.

### Phase 1: Building FQHC capacity for “descriptive” operational research

In 2017, the FQHCs and MITRE recognized the need to collect data that could inform operational strategies, and the opportunity to collaborate to pool data across the FQHCs, enabling analysis as well as comparison across the health centers. Known as the “Minimum Common Metrics,” (MCM) the FQHCs gathered anonymous data from their research participants (and potential participants) that could be summarized to look for patterns in areas such as motivation to join or decline, as well as operational considerations such as the time required to complete enrollment elements [9].

MITRE supported the FQHCs by periodically examining the MCM data and summarizing notable observations, as well as by providing forums for the FQHCs to share their own interpretation of patterns in the data. Additionally, as FQHCs learned more about their own cohorts and the effective management of their research operations as evidenced by the MCM and program data, they began looking for opportunities to share their insights externally. For example, FQHC authors created posters for presentations at regional and national meetings to share their learnings and particularly about how they may relate to research being undertaken in similar communities and settings, such as by other FQHCs.

As the FQHCs continued to enroll more participants and the program continued to mature, the pool of available data as well as the potential data inquiries also expanded. It was at this point that FQHCs began pursuing manuscript publication in addition to ongoing dissemination via posters and presentations. However, these manuscripts were still limited to the arena of descriptive operational research, which largely made use of operational and custom survey data. For example, as the program began planning to return genetic results to *All of Us* participants, one of the FQHCs described primary care providers’ readiness for delivering genetic services in an FQHC environment [10]. Similarly, as the program initiated the capability for *All of Us* participants to contribute data from their fitness tracker devices, another FQHC presented findings from a survey of FQHC patients about their use of fitness tracking devices and potential barriers and facilitators to increased use [11].

### Phase 2: Building FQHC capacity for “Exploratory” operational research

The evolution of the FQHC research landscape took a major leap when *All of Us* launched special initiatives geared towards testing new strategies for operational improvements. These initiatives allowed FQHCs to hypothesis test their strategies to gain insights on the successful ones that worked well and learn from those that did not work. These insights not only allowed FQHCs to continue presenting these topics through posters/presentations at conferences, but they also culminated in the development of ideas for potential manuscripts for submission to peer-reviewed journals. A summary of special initiatives that were relevant in shaping the evolution of the FQHC research landscape are provided in the sections below.

#### Incubators

Incubators allowed FQHCs to develop and test new strategies related to engagement, enrollment, and retention. The incubator framework included a stepwise process of ideation, testing, and evaluation, all supported by MITRE expertise in research design and data analysis. The first incubator project tested a new method for transporting biospecimens between enrollment sites; a second incubator initiative focused on FQHCs testing strategies and processes to enroll individuals who did not receive care at their respective health centers, whereas previously *All of Us* FQHCs only enrolled their own patients.

#### Enrollment/Retention pilots

Enrollment/Retention pilots focused on testing strategies for enrolling and retaining participants from their communities, expanding the *All of Us* reach into new geographic areas and/or accelerating survey completions. A total of seven pilots proposed by FQHCs were approved to move forward. Three pilots focused on utilizing a temporary structure as an alternative space for enrolling participants to expand the geographic reach of the potential participant pool, two pilots focused on testing incentives for enrollment and/or retention, one pilot focused on the use of community health workers to increase enrollment and enrollment/retention referrals, and one pilot tested the use of a ride share service to reduce transportation barriers for participant enrollment at the FQHC.

#### Pediatric readiness assessment

In Fall 2023, the very first pediatric participants were enrolled in *All of Us*. Given the sensitive nature of enrolling children into a longitudinal study, the program took a conservative approach through small-scale testing by first inviting a small number of enrollment partners, including two FQHCs, to participate in a readiness assessment. Protocols, technology, and communication was first tested with a handful of pediatric participants and their parents and made refinements, as needed, to make sure it worked in different environments. The participating FQHCs adapted the pediatric protocol for their specific settings and to ensure that children and families from a broad range of backgrounds were able to participate. As they implemented the pediatric protocol, the FQHCs documented and shared observations and lessons learned which could ultimately inform enhancement and modifications prior to a larger-scale rollout of pediatric participation in the future.

#### Formalized launch of FQHC publication guidelines

The operational research work and ideas generated from the initiatives led to the development of publications guidelines as a formal way for FQHCs to propose their ideas to MITRE and the NIH. The form was used by MITRE and NIH to assess, refine and approve FQHC publication ideas. Following this deliberation, the FQHC(s) submitted the idea to the *All of Us* publications board for approval. In many cases, FQHCs collaborated with MITRE research staff on manuscript development. For example, a paper on understanding the association between *All of Us* FQHC participants’ digital readiness and their preferred mode of *All of Us* survey completion included authors from MITRE and FQHC [12].

#### Python training

To enhance the FQHC analyst staff knowledge, MITRE developed training materials that covered the fundamentals of using the *All of Us* RW, Jupyter Notebooks, and Python to interact with data from the *All of Us* cohort. The goal of Python training was to teach FQHC staff who have some familiarity with programing and experience analyzing data to carry out familiar tasks using the tools available in RW. Training included five lessons on the following topics:

- Lesson 1. How to use the SQL builder and pull a data frame into Jupyter
- Lesson 2. How to manipulate data
- Lesson 3. How to visualize data
- Lesson 4. How to run a basic statistical model
- Lesson 5. Pulling it all together. An example using all the above

### Phase 3: Moving FQHCs toward biomedical research through collaboration

As the FQHCs focused on products to further promote and develop the operational aspects of their research, *All of Us* supported the advancement of data quality and research enhancement through collaborations with RMCs and MITRE, both of which have the infrastructure and staff capacity for research and analytics.

NIH staff reached out to large RMCs and *All of Us* FQHCs to determine interest level for developing collaborative research projects using the *All of Us* data. Once this was established, a meeting was conducted to establish roles and responsibilities, timelines, and expected products. After these initial discussions, NIH staff requested that partners report back to the program on the progress of these collaborations in consortium meetings.

The program also supported the advancement of research and data quality using the “demonstration project” and “driver project” mechanisms. These projects aim to maximize impactful use and drive scientific innovation of the data by evaluating and advancing data quality and analytic insights through targeted research questions of value to the scientific and medical community. To facilitate this critical work, the program provides supplemental funding to various partners to conduct these studies. Partners without the staff or skills, or the infrastructure needed to lead research, like many of the FQHCs, may also collaborate with others equipped with the required resources to help foster the development of these capabilities more broadly.

The FQHCs leveraged MITRE’s research infrastructure given the management model through the health FFRDC. MITRE provided staff with analytics and research knowledge to aid FQHCs in conducting these projects. Specifically, MITRE helped FQHCs delineate whether the data available in the RW would support research topics of interest and provided publication and statistical analyses expertise to aid in producing two draft manuscripts for research journal submission. The next section provides more details on the FQHC driver project, a use case of building FQHC biomedical research capacity and advancing the *All of Us* data quality through collaboration.

#### FQHC driver project

The FQHC driver project team (team) was geographically dispersed across the contiguous United States, including three FQHCs (Table 2): San Ysidro Health (SYH), based in San Diego California, Moses/Weitzman Health System’s Community Health Center, Inc. (CHCI) based in Middletown Connecticut, and Cherokee Health Systems (CHS) based in Knoxville Tennessee. Team members from each FQHC included one required Clinical Subject Matter Expert (SME) and an optional data analyst from each FQHC to shadow and learn how to conduct analyses in the RW.

**Table 2.** Sample characteristics of Federally Qualified Health Centers (FQHCs) participating in driver projects[13]

	Service locations (excluding schools)	Patient volume, 2023	Patients at/below 200% of federal poverty guideline	Patients best served in language other than english	Patients experiencing homelessness
Community Health Center, Inc.	53	107,225	89.2%	32.0%	2.5%
San Ysidro health	44	128,158	90.2%	37.9%	3.2%
Cherokee health systems	20	65, 962	91.4%	16.2%	10.6%

#### Timeline and collaboration model

**Figure 2.**
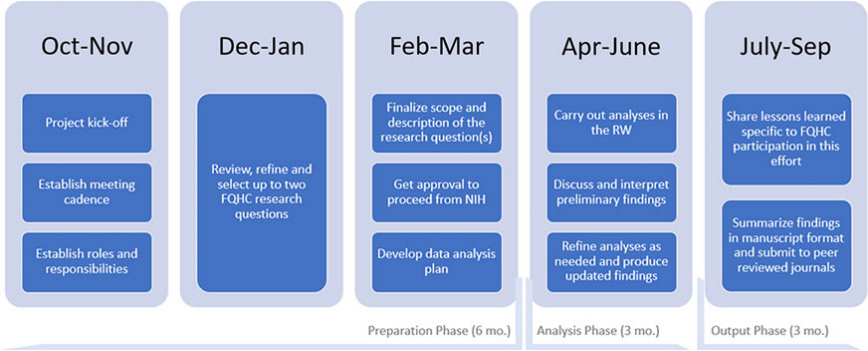
Project timeline.

The collaboration followed a remote model, with artifacts shared across the team through shared drive. Regular meetings and resources facilitated knowledge exchange and project execution; and while MITRE facilitated the meetings, the team shared note-taking responsibilities. The overall project timeline is shown in Figure 2 and consists of preparation, analysis and output phases. Each phase is described in more detail in the sections below.

#### Preparation phase

The preparation phase involved aligning the team on the driver project objectives and brainstorming research questions. During this phase, the team met every two weeks for one hour. A total of six questions were initially proposed across the three FQHC teams, out of which two were selected to move forward, described below. The intended selection goal was to choose research questions that were likely to resonate with FQHC patients and participants.

- How are social determinants of health and genetic factors associated with the severity of Type 2 diabetes in a broad spectrum of backgrounds?
- What is the experience with healthcare services among females with Type 2 diabetes who have been less well studied in biomedical research?

The first research question was important to SYH and CHCI, where Type 2 diabetes disproportionately affects their patient population [14]. Therefore, research question 1 directly aligned with the health priorities of the two FQHCs. The second research question, proposed by CHS, was an extension of an existing research study focused on addressing how outcomes are differentially impacted by health determinants. This study, funded by the Robert Wood Johnson Foundation, included 50 participants across three CHS clinic sites. CHS proposed this research question as an opportunity to scale up the study by using a larger sample of participants and a larger geographical area in the *All of Us* data.

#### Analysis phase

The analysis phase began with brainstorming to identify relevant variables of interest to achieve the objectives of each research question. The team met every two weeks and key activities included conducting literature reviews, developing the requirements for the study population of interest and selection of variables for further analysis. Towards the end of this phase, the team also developed a manuscript template and identified target journals for submission.

#### Output phase

In this phase, the analysis was finalized, and team members were assigned writing responsibilities on the manuscript. In addition, during this phase, each FQHC team member submitted a document titled “FQHC Lessons Learned from the Driver Project Participation” which focused on capturing lessons learned in the areas of research question development, RW experience, team meeting structure, data analysis discussions, manuscript development experience, and overall project organization. Recommendations for replicating biomedical research with FQHCs in the future are summarized in the next section, based on the lessons learned from the FQHC driver project.

### Recommendations for replicating biomedical research collaboration with FQHCs

The FQHC driver project was a valuable learning experience for FQHCs, providing a deeper understanding of the RW and its functionalities, and underscored the significance of understanding data availability while formulating research questions. Recommendations for replicating biomedical research collaboration with FQHCs are included below. We note that many of these recommendations reference staff roles that may not exist at some FQHCs, or that may not have time/funding to contribute the necessary effort; these cases highlight the importance of partner organizations such as collaboration with universities and other local sources of expertise.

- Utilize the project plan and meeting cadence described in this document for engaging FQHCs.
- Assign a person or people to project manager and data scientist roles. Plan for about 1 FTE for 2 – 3 driver projects. FTE requirements are expected to be higher on projects that require genomic analysis.
- Involve FQHC clinical SMEs who can dedicate their time to research and balance competing engagements (Alternatively, identify subject matter experts from partner organizations).
- Invest in training FQHCs on coding tools (Python, SAS) and helping get familiar with relational databases for interacting with EHR to enable FQHCs to conduct deeper analyses beyond the point and click tools (Alternatively, identify required expertise from partner organizations).
- Maintain a small team of data scientist SMEs, who may be in-house or drawn from partner organizations, with relevant expertise that could either work with or provide guidance from the inception of the project, including guidance on initial research questions and potential study designs.
- Clarify roles and responsibilities early and revisit them often to ensure accountability and ownership of the project.

## Discussion

As *All of Us* continues to ensure that the focus on precision medicine is reflected in both the data collected and the research conducted, continuing to advance the research landscape in FQHCs is crucial. In addition, the FQHC driver project topics were relevant to FQHCs and their communities. This is important so that resulting biomedical research discoveries and breakthroughs are applicable to all populations.

Lessons learned from the projects exemplified in this manuscript could be used to continue to meet this goal. Setting up collaborations between partners is optimal to bring together variable expertise such as subject matter expertise, knowledge of local community health needs, research publication, and statistical expertise. This can also be leveraged by using tools available in RW that were developed to allow for collaboration. Over time, some health centers may choose to grow their investment in in-house expertise to support research activities, while for others, we acknowledge that building a robust internal research infrastructure may not align with overall health center priorities or funding scenarios, particularly in an environment of limited resources when the main focus is understandably on patient care. The latter case underscores the importance of FQHCs being able to leverage their strong relationships in the community, such as partnering with subject matter experts from a local university or public health department.

For the driver projects, the FQHCs could leverage expertise and staff from MITRE. As noted earlier, for FQHCs to conduct these projects independently and without collaboration with other larger research entities, research staff with data analytic skills would be needed to support subject matter expertise inherent in clinical settings. This would facilitate not only greater research capabilities within the FQHCs but would also foster a deeper understanding of the potential use and impact of the data collected and available for research relevant to their unique communities and needs. This further illustrates that building an enduring collaborative capacity, where FQHCs are equipped to be effective partners in research networks, may be a more impactful model for some FQHCs to adopt, while other FQHCs may indeed have the capacity to operate as fully independent research centers.

Additionally, building a strong research foundation through projects and topics relevant to FQHCs and their communities will evolve with each new data release in RW. A rich range of biomedical data is already available for research use, including EHRs; physical activity, heart rate, and sleep data from wearable devices; genomic data; physical measurements on height and weight; survey data capturing important demographic, lifestyle, and socioeconomic factors; and more. And with each new data release, additional data and data types will be added, increasing the potential for precision medicine research for a broad range of populations. Upcoming data releases as highlighted in the recently published Data Roadmap (https://allof-us.org/Roadmap) will feature greater numbers of participants, additional populations groups of interest, richer genetic data, participant-mediated EHR data, claims data, and more. Thus, by building this capacity within the FQHCs today, we are developing a research infrastructure that will continue to grow along with the *All of Us* data, supporting ongoing advancements in precision-based medicine that can result in improved health outcomes. The next section provides perspectives from the two FQHCs (CHCI and SYH) that participated in the driver project efforts about their future vision and how the driver project contributed to that vision.

### FQHC (CHCI and SYH) perspectives on future vision

CHCI is committed to strengthening its internal capacity for public health genomics research. This includes maximizing access to *All of Us* data, refining staff skills in using RW, and deepening partnerships with NIH, MITRE, other FQHCs, and the public health genomics research community. Through *All of Us*, CHCI is better able to identify capability gaps and areas where CHCI should continue to make investments (e.g. enhancing staff proficiency in Python for genomic data analysis). Additionally, CHCI seeks to build on existing infrastructure and collaborations to pursue new funding opportunities and expand research efforts. Finally, a key priority is ensuring that research findings are effectively translated into FQHC settings, strengthening the connection between research, clinical providers, and patients.

SYH’s participation in *All of Us* fostered valuable experience and developed new capabilities related to the unique environment of the RW. The collaborative framework enabled SYH to expand its research efforts and gain a deeper understanding of the complexities associated with accessing, cleaning, and analyzing data within RW. This experience proved particularly valuable given the complexity of the RW, accelerating SYH’s learning curve in navigating the intricate process of accessing and utilizing RW data. This challenge is not unique to FQHCs, as academic and industry researchers alike require dedicated training to effectively utilize RW. The collaborative framework, combined with the support of bioinformaticians and biostatisticians, enabled SYH to bridge gaps in internal expertise typically filled by academic partners and laid the groundwork for future collaborations. While SYH had infrastructure, experience, and capacity in place, these projects are contributing to the expansion of its research infrastructure. Moving forward, SYH aims to continue exploring the capabilities of RW, including new data releases and advanced analytics tools, to address critical research questions impacting the health of the communities served by SYH.

## Conclusions

This paper described the evolution of research landscape of MITRE-managed FQHCs, from building capacity for descriptive, to exploratory operational research, and moving toward biomedical research. Although some *All of Us* FQHCs started the program with more advanced research capabilities than others, the paper demonstrated that building an enduring collaborative capacity, where FQHCs are equipped to be effective partners in research networks, is an appropriate and impactful model for some FQHCs to adopt, while other FQHCs may be fully capable of standing up independent research centers.

By developing this capacity, we are developing a research infrastructure that will continue to grow, supporting advancements in precision medicine for improving health outcomes. Lessons learned from the projects and collaboration models exemplified in this manuscript highlight the importance of biomedical research discoveries applicable to all populations, and the importance of fostering a deeper understanding of the potential use and impact of the *All of Us* data collected and available for research relevant to unique communities and needs.
